# Assessing the Phenotype of a Homologous Recombination Deficiency Using High Resolution Array-Based Comparative Genome Hybridization in Ovarian Cancer

**DOI:** 10.3390/ijms242417467

**Published:** 2023-12-14

**Authors:** Svetlana Magadeeva, Xueqian Qian, Nadine Korff, Inken Flörkemeier, Nina Hedemann, Christoph Rogmans, Michael Forster, Norbert Arnold, Nicolai Maass, Dirk O. Bauerschlag, Jörg P. Weimer

**Affiliations:** 1 Department of Gynaecology and Obstetrics, Christian-Albrechts-University and University Medical Center Schleswig-Holstein, 24105 Kiel, Germany; 2 Institute of Clinical Molecular Biology, Christian-Albrechts-University and University Medical Center Schleswig-Holstein, 24105 Kiel, Germany

**Keywords:** ovarian cancer, HRD, PARP-inhibitor, aCGH, genomic scars

## Abstract

Ovarian cancer (OC) cells with homologous recombination deficiency (HRD) accumulate genomic scars (LST, TAI, and LOH) over a value of 42 in sum. PARP inhibitors can treat OC with HRD. The detection of HRD can be done directly by imaging these genomic scars, or indirectly by detecting mutations in the genes involved in HR. We show that HRD detection is also possible using high-resolution aCGH. A total of 30 OCs were analyzed retrospectively with high-resolution arrays as a test set and 19 OCs prospectively as a validation set. Mutation analysis was performed by HBOC TruRisk V2 panel to detect HR-relevant mutations. CNVs were clustered with respect to the involved HR genes versus the OC cases. In prospective validation, the HRD status determined by aCGH was compared with external HRD assessments. Two *BRCA* mutation carriers did not have HRD. OC could approximately differentiate into two groups with characteristic CNV patterns with different survival rates. Mutation frequencies have a linear regression on the HRD score. Mutations in individual HR-relevant genes do not always indicate HRD. This may depend on the mutation frequency in tumor cells. The aCGH shows the genomic scars of an HRD inexpensively and directly.

## 1. Introduction

Ovarian cancer (OC) is the gynecological tumor with the statistically poorest prognosis [1]. Similar to many solid tumors, it is characterized by pervasive chromosomal changes. The chromothripsis results from an ineffective repair capacity of the cells [2,3]. Cells have different DNA repair pathways [4]. DNA single-strand breaks are recognized by control mechanisms in the replication forks and repaired by base excision repair (BER) [5,6,7]. Among other enzymes, poly(ADP-ribose) polymerase 1 (PARP) is involved in this [8]. If they fail, single-strand breaks can be repaired by the homologous recombination (HR) system [9], which additionally repairs double-strand breaks [10]. The DNA damage detection complex includes BRCA1 which organizes the *BRCA1*-associated genome surveillance complex (BASC) [11,12]. The homologous repair mechanism is a multigenic process involving many genes. Most of these genes are also involved in HR. Typical genes involved in the initiating MRN complex are *MRE*, *RAD50*, and *NBS1* [13]. *BRCA2*, *BALB2*, *RAD51*, and *RAD54* connect to homologous regions of sister chromatids [14,15]. In particular, if a gene mutation damages HR and impairs its functions, the cell is left with the only repair pathway available—a single-strand repair mechanism [16]. Errors in the HR system can lead to cross-over and chromosomal substitutions with other non-homologous chromosomes. These exchanges result from a non-homologous repair (NHEJ and MMEJ) that can link breakpoints on different chromosomes [17]. Overall, tumor cells with a deficit of homologous recombination (HRD) accumulate larger imbalances due to migrating cross-over structures of non-homologous chromosomes. Subsequent mitotic cell divisions of cells with translocated chromosomes can also cause chromosome portions to be lost or multiplied, leading to genomic gains or losses [18,19]. This results in “genomic scars” on the chromosomes, such as large-scale transitions (LST), which are defined by recognizable breakpoints larger than 10 Mb apart [20]. The translocated chromosomes that contribute to genomic gains or losses greater than 11 Mb and extend to the chromosome ends are described as telomeric allelic imbalances (TAI) [18]. If the mitotic distribution of translocated chromosomes leads to the loss of maternal or paternal chromosome parts, this results in a loss of heterozygosity (LOH). LOHs over 15 Mb in length are considered in HRD score determination. The summation of these genomic scars, consisting of LOHs, TAIs, and the breakpoints of LST, results in the overall/total HRD score [20]. It is known that the resulting CNVs have an influence on the expression of tumor-relevant genes [21,22].

Promising treatment options for OC have emerged in recent years due to the availability of PARP inhibitors [16]. Clinical studies have shown that patients with a mutation in the *BRCA* genes benefit significantly from PARP inhibition [23,24,25,26]. Drugs, such as PARP inhibitors, can specifically prevent single-strand repair in the cells [27,28]. A PARP inhibitor deprives the HR-deficient cell of the last possible alternative to repair single-strand breaks. The drug hinders the polymerase in single-strand repair and makes it inoperable, thereby forcing the cell to die. Thus, cells with an HRD lack a redundant system for repairing single-strand breaks. If a regular repair system for single-strand breaks also fails in cells with an HRD, the replication forks stop at the DNA defect and the cells arrest in the S-phase [29,30]. Finally, the cells perish through programmed cell death [31]. To achieve the goal of destroying HRD tumor cells, it is essential to determine to what extent tumor cells have HRD. In addition to mutations in *BRCA1* and *BRCA2*, HRD can also be induced by defects in other genes involved in HR. If the tumor cells do not show any HRD, treatment with PARP inhibitors is less effective [32,33]. The effect of a PARP inhibitor would be reversed by repair involving HR, thereby conferring resistance to the PARP inhibitor on tumor cells.

Whether tumor cells have an HRD will help utilize PARP inhibitors therapeutically effective in ovarian carcinoma patients. Mutation analyses alone do not lead a reliable HRD diagnosis, as 95% of tumors with a *BRCA* mutation have an HRD [34]. With array-based comparative genome hybridization (aCGH), it is hardly possible to identify destructive mutations in the genes involved in HR. However, the aCGH can successfully identify the genomic scars that make up the substantive phenotype of an HRD, based on the criteria developed by Timms et al., 2014 [20]. The summation of genomic aberrations, such as the number of breakpoints by LST, the number of TAI, and LOH events, is reflected in the cytogenetic phenotype of an HRD, if the sum of the genomic scars found in a tumor is 42 or over. There are legitimate reasons to lower the recognized HRD limit from 42 to the mid-30s [35]. However, since the meaningful studies on the effectiveness of PARPi in OC patients with HRD used the cut-off value of 42, and this has so far been used in the assessment of HRD, this value should also be used when comparing high-resolution aCGH. A frequent but also indirect method is the interpolation of this phenotype by new generation sequencing (NGS) and complex single nucleotide polymorphism (SNP) analyses of HR involving genes. There are also SNP arrays from Affymetrix, or the Myriad MyChoice test, directly determining the HRD status as a phenotype. Furthermore, there are efforts to derive the genomic scars from which the HRD phenotype can be inferred by biostatistical genome reconstruction from the NGS data, as done in the NOGGO GIS v1 Assay [36]. Here, we show that HRD phenotype detection in ovarian carcinoma is also possible by using high resolution array platforms, bypassing complex NGS methods, or unique SNP arrays and genome reconstruction. A high-resolution aCGH with sufficient SNP information is technically ideal for detecting larger chromosomal aberrations and LOHs, as is necessary to detect HRD. While aCGH directly maps these values comprehensively across the genome, genome-wide NGS methods first have to reconstruct them with great effort or only map genes involved in HR. The economic advantages of a cost-effective aCGH compared to a complex and expensive HRD test for a treatment decision are obvious.

## 2. Results

### 2.1. Determination of HRD Score Cut-Off

Of the 30 ovarian carcinomas from the test cohort, 6 showed a mutation in *BRCA1* and 5 in *BRCA2*. These 11 OC cases with mutations in *BRCA1* and *BRCA2* served as positive controls. Determination of the HRD limit value from tumors with a mutation in *BRCA1* or *BRCA2* resulted in a cut-off HRD score of 35 (Table 1). 

Considering the relatively small sample size, this cut-off is relatively close to the now validated and internationally recognized value of 42 [23,34]. Nine of 11 tumors with a mutation in *BRCA1* or *BRCA2* had an HRD score of over 42 and ten over 35. In the case of the tumor 16-P-38, despite a *BRCA2* mutation with a very different HRD score of 7, reducing the cut-off we determined, we decided to use the commonly used cut-off of 42. 

### 2.2. Panel Sequencing and HRD-Score

Among the 30 OC examined, at least 1 mutation in *TP53* was also present in 22 ovarian carcinomas (Table 2). Further mutations could be detected in 14 cases in the genes *CHEK2*, *MLH1*, *MUTYH*, *SLX4*, *RECQL*, *RAD51D*, *RAD51C*, *NF1*, *Fam175A*, *FANCM*, *CDH1*, *PMS2*, *MSH2*, and *MSH6* (Table 2). In 10 cases these mutations were associated with *BRCA1*, or *BRCA2* and *TP53* mutations. All of these tumors had an HRD score of 36 to 150 and are thus above the HRD cut-off limit—except for case 72-S-59. All 19 HRD-positive ovarian carcinomas had a mutation in *TP53*. Three tumors with a mutation in *TP53* do not meet the criteria for an HRD phenotype. Case 72-S-59, with an additional mutation in *BRCA1*, was close to the cut-off point with an HRD score of 36.

**Figure 1:**
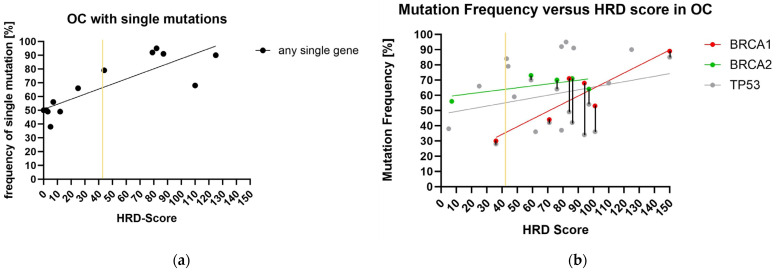
(**a**) Regression analysis of the mutation frequency of all cases with only one detected mutation and the corresponding HRD score. The regression line shows a direct dependence of the mutation frequency on the determined HRD score (R^2^ = 0.7; *p* = 0.0004). (**b**) Shown here are the regression lines of the mutation frequencies of *BRCA1*, *BRCA2*, and *TP53* in relation to the corresponding HRD scores. Red = regression line *BRCA1* (R^2^ = 0.8; *p* = 0.015); green = regression line *BRCA2* (R^2^ = 0.41; *p* = 0.25); gray = regression line *TP53* (R^2^ = 0.07; *p* = 0.23). Vertically connected dots correspond to multiple mutations in one case. The yellow lines correspond to the HRD limit of 42.

The *BRCA1* mutation in this tumor (72-S-59) had a 30% frequency and an additional mutation in *TP53* (frequency = 28%) and in PMS2 (frequency = 47%). However, the HRD-negative tumor 16-P-38 with a mutation in *BRCA2* (frequency = 56%) had no further mutations in *TP53* or other genes. Of the 19 ovarian carcinomas with proven HRD, 14 tumors had multiple mutations. Of the tumors without the HRD phenotype, the indistinct tumor 72-S-59 showed multiple mutations. However, these five HRD-positive tumors with one mutation in *TP53* had relatively high mutation frequencies of 68% to 95% (mean = 85%). The seven individual mutations in HRD-negative tumors had a mutation frequency of 49% to 66% (mean value = 51%) (Figure 1). The mutation frequency of all cases with just one detected mutation is compared with the corresponding HRD score in a regression analysis in Figure 1a. This results in a direct dependence of the mutation frequency on the determined HRD score (R^2^ = 0.7; *p* = 0.0004). In Figure 1b, the mutation frequencies are plotted against the HRD score of all investigated cases with *BRCA1*, *BRCA2*, or *TP53* mutations separately. The frequency of mutations in all three genes tends to increase with the HRD score. A regression of the mutation frequency against the HRD score revealed a confirmed increase in the HRD score with an increase in the mutation frequency in *BRCA1* only (*BRCA1*: R^2^ = 0.80, *p* = 0.01). No correlation between HRD and mutation frequency could be derived for *TP53* and *BRCA2* (*TP53*: R = 0.07; *p* = 0.23; *BRCA2*: R = 0.41, *p* = 0.25).

### 2.3. CNV Cluster Analysis in 132 Tumor Genes and 30 OC Cases

**Figure 2:**
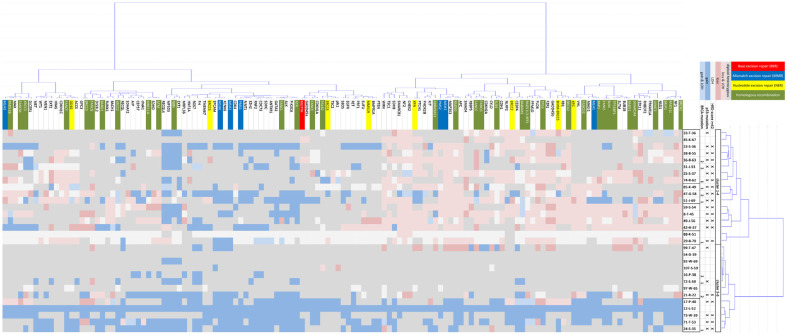
Biaxial clustering of 132 tumor-related genes in 30 OCs with respect to their genomic gains, losses, and LOHs. The genes with homologous recombination (HR) functions are highlighted in green. Genes involved in nucleotide excision repair (NER) are highlighted in yellow. Genes that function in mismatch excision repair (MMR) are highlighted in blue, and genes that function in base excision repair (BER) are highlighted in red. In the cluster matrix, gains are marked in blue and losses are highlighted in red. LOHs are shown in a lighter color, and an unremarkable diploid state is shown in grey. The OC cases were sorted into the clusters 1–4 group and the clusters 5–6 group according to the cluster tree.

We subjected the 132 tumor genes over- or under-represented in conspicuous CNVs and LOHs in the 30 OC cases to biaxial cluster analysis (Figure 2). The 30 OC cases differentiate their CNV patterns into 2 summarized clusters, which are composed of sub-clusters 1–4 on the one hand and sub-clusters 5 and 6 on the other. It is striking that in clusters 1–4, 82.35% (14/17) of the OC have an HRD and a mutation in *TP53*. In clusters 5–6, this is only 38.46% (5/13) of OC. The proportion of mutations in the *BRCA* genes is also higher in clusters 1–4, with 41.18% (7/17), than in clusters 5–6, with 30.77% (4/13). 

**Figure 3:**
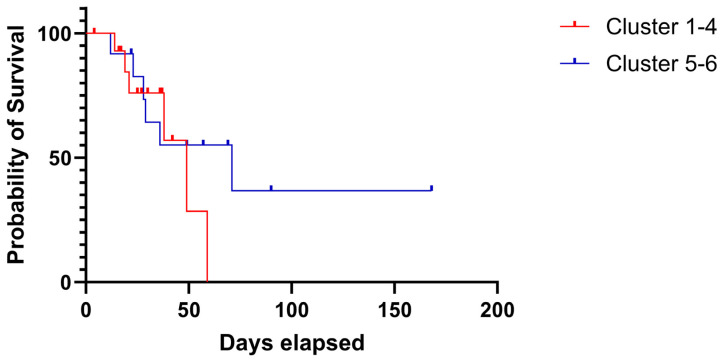
Kaplan–Meier curve of OC cluster groups 1–4 (red) and cluster groups 5–6 (blue). The mean survival of cluster groups 1–4 is 59 days, well below the mean survival of 168 days of cluster groups 5–6. A significant difference between the two-course curves could not be determined (*p* = 0.32).

The clustering gains and losses, and the LOHs show broadly comparable patterns within the clusters. While the CNV pattern in clusters 5–6 is almost the same across most genes, the OC case in clusters 1–4 shows a gene cluster with predominantly losses from the *BRCA1* gene to the *WRN* gene, a gene cluster with predominantly gains from the *PTEN* gene to the gene *RECOL4*, and another gene cluster with a more heterogeneous CNV pattern from gene *ATM* to gene *MLH3*. The survival rates of the patients in both summarized cluster groups were compared. The median survival of patients in clusters 1–4 is 59 days, while patients in clusters 5–6 survive a median of 168 days. Although the Kaplan–Meier curves (Figure 3) suggest an advantage for clusters 5–6, the difference between the two survival curves is not significant (*p* = 0.32), most likely because of the small sample sizes.

### 2.4. CNV Differences in 30 OC Cases with and without BRCA Mutation

The most significant CNV differences between OCs with mutations in the *BRCA* genes and those without *BRCA* mutations are in chromosome 10p15.3 (chr10:171262–1306517) (Table 3). While 15/19 (78.95%) OCs without *BRCA* mutations in this region have no CNV, only 3/11 (27.27%) of OCs with *BRCA* mutations are balanced, whereas 8/11 (72.72%) OCs with *BRCA* mutations at this position have genomic gains. OC without *BRCA* mutations only has 3/19 (15.79%) gains here. This difference in genomic dose between OC with *BRCA* mutation carriers and OC without *BRCA* mutation carriers is significant with a Chi^2^ = 0.0073. However, there are no relevant genes in this region that are known to influence tumor development. Another slightly less significant difference between OCs with and without *BRCA* mutations is found in 11p15.4 (chr11:5785900–5809417). Here, 7/11 (63.63%) OCs with a *BRCA* mutation in the tumor show a higher percentage of losses than the OC cases without a *BRCA* mutation in 2/19 (10.53%). This difference is also very safe with a Chi^2^ value of 0.008. There are no known tumor-relevant genes in this region either.

### 2.5. Validation of HRD Detection Using aCGH on 19 OC

In the validation, we examined 19 tumors with known HRD status using 4 × 180 k CGH+SNP microarrays (Table 4). For 15 cases, we were able to confirm the HRD status (which had been externally checked) using Agilent microarrays with a cut-off HRD score of 42.

**Figure 4:**
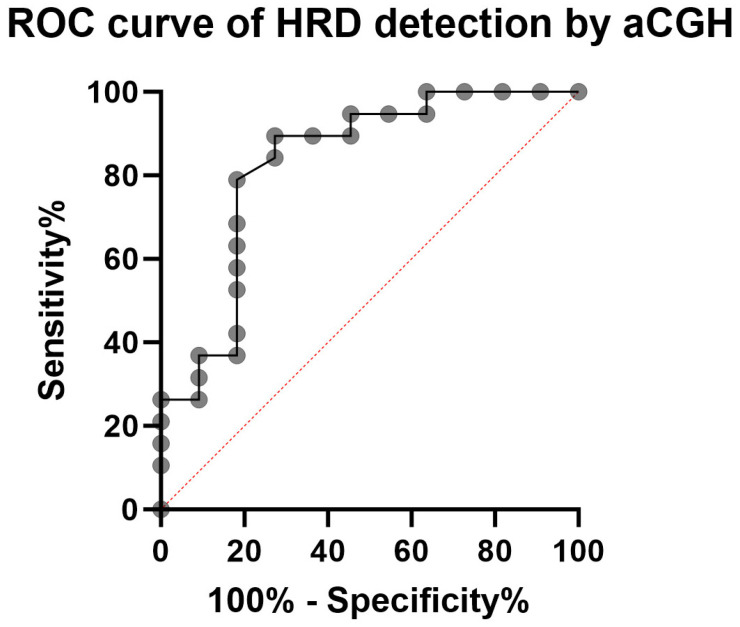
ROC curve (Receiver Operating Characteristics) of HRD detection by high resolution aCGH. The sensitivity is 0.77, and the specificity is 0.8 using 4 × 180 k arrays from Agilent. The area under the curve is 0.8301 (*p*-value = 0.003).

Two cases showed no mutations in *BRCA1/2* but still had HRD by aCGH. Two cases did not meet the criteria for an HRD phenotype despite mutations in the *BRCA* genes. However, one of the two *BRCA* mutation carriers (Valid-11), with an HRD score of 41, remained just below the cut-off point. This resulted in a sensitivity of 0.77 and a specificity of 0.8 for the high-resolution aCGH test using 4 × 180 k arrays from Agilent. The cases in Table 1 with confirmed *BRCA* genes mutations can be considered positive controls. If one compares the patients in the validation (Table 4), then the Receiver-Operating-Characteristics (ROC) curve (Figure 4) shows an area under the curve of a respectable 0.8301 (*p*-value = 0.003). 

## 3. Discussion

Since 2016, the detection of the phenotype of a deficit in homologous recombination (HRD) in tumors has been protected by a patent from Myriad (US 9,388,472 B2). In particular, this patent protects the minimum length of a detected LOH, which is used to calculate an HRD score. With MyChoice, Myriad offers a commercial test for recording the HRD. Another company (Affymetrix) offers an SNP array that allows HRD recording. The Northeast German Society for Gynecologic Oncology (NOGGO) has also published a test to determine HRD (NOGGO GIS v1 Assay) [36]. This test is based on a panel sequencing of the *BRCA* genes and 55 genes relevant to homologous recombination repair (HRR). Furthermore, this test collects information about 20,000 single nucleotide polymorphisms (SNPs) distributed across the genome. The proportion of LOHs necessary to collect the HRD score can be determined on this basis. However, whether this procedure affects Myriad’s patent interests may be questionable. To phenotypically detect HRD, representative and comprehensive information about genomic imbalances and LOHs is necessary. There is no doubt that the techniques mentioned here meet this requirement. However, we show that high-resolution aCGH can also display the HRD phenotype at a much cheaper cost.

We were able to detect the genomic scars of a case to determine an HRD score for just under EUR 300 using high-resolution aCGH, although without taking personnel costs into account. In 2019, the costs for a multigene panel analysis were between USD 3711 and USD 4796 [37]. In 2020, the costs for targeted gene panel sequencing fell to EUR 1000 [38]. Corresponding HRD tests for clinical diagnostics usually exceed these costs. However, a clinician may only use these approved and verified tests to make treatment decisions. It would be good if alternative procedures such as the high-resolution aCGH presented here contributed to price competition after testing and approval in larger studies. It is becoming increasingly clear that NGS panel sequencing of HR-relevant genes alone, including the *BRCA* genes, is insufficient to derive the HRD phenotype. Our data confirm this. Alterations in the HRR genes can only derive an HRD by interpolation. Even a negative finding of such panel tests does not rule out an HRD in general [34]. It is possible that deletions of individual exons of genes involved in HR or epigenetic changes cannot be detected. This HRD diagnostic reveals the dilemma of global healthcare systems characterized by market economies. Clearly, a company protects its interests through patent law and forces the use of the protected test systems. As a result, this encourages the development of other diagnostic tools that requires additional funding and resources to circumvent patent protection. However, if the results are less accurate or the tests are significantly more expensive, money that is urgently needed elsewhere in the healthcare system is lost. There is evidence that the display of an HRD phenotype instead of an HRD score may additionally be replaced by alternative indices [39,40]. TMB is increasingly being considered as an index of repair insufficiency [41]. However, there are still no clear, reliable studies and comparisons to valid HRD testing for an index application to make a therapy decision. Index surveys must be compared with validly collected HRD recordings in large-scale studies in order to be able to make a statement about the usability of such biomarkers. Our data do not reveal any correlative regression between HRD score and TMB index (Supplementary Table S1). It is quite conceivable that another form of an index that reflects HR-relevant mutations correlates with the usability of PARPi. This would have the advantage that Myrriad’s patent protection would also remain unaffected. However, we have to admit at this point that the sample size of only 30 OCs examined is far too small to be able to make valid statements about this. The detection of the true therapy-indicating phenotype HRD remains the most reliable method for the patient. Regardless of the patent situation and considering the definition of an HRD phenotype, we could detect genomic scars without very expensive commercial test systems. Simple high-resolution microarray application was relatively cheaper and faster to carry out. Obtaining this information is what a physician needs for a successful choice of therapy. However, evaluating the significantly cheaper high-resolution aCGH may also affect Myriad’s patented method for LOH assessment. Evaluating this should not be our goal at this point.

The composition of our microarrays differs from commercial ones only in that we have increased the design for high resolution density of samples in *BRCA1* and *BRCA2* genes. The OC cases listed in Table 1 all had mutations in the *BRCA1* and *BRCA2* genes detected by panel analysis. However, since not all *BRCA* mutation-carrying tumors also have an HRD [34], it is not certain whether all 11 positive control cases (Table 1) are genuinely HRD-positive. Thus, whether these can be used entirely as a positive control is questionable. In this positive control there may well be tumors in which the HR system still functions to such an extent that the phenotype of an HRD does not form, despite the *BRCA* mutation. As previously published studies showed, only 95% of cases with *BRCA* mutations have HRD [34]. The cut-off we determined for an HRD score, above which we can assume the HRD phenotype, is 35. However, with 11 cases, this sample size is very small. OC cases without the HRD phenotype would bias the cut-off down in this group of positive controls. The cut-off determined by us is, in fact, below the limit value of ≥42, which is used when using the MyChoice test from the company Myriad or other international HRD tests. Since our aCGH analysis uses the same criteria as the summation of LST breakpoints, TAI, and LOH to determine an HRD, it is quite permissible to apply the same limit value to high-resolution aCGH. However, our positive control with cases 72-S-59 and 16-P-38 would be biased in this case. For further evaluation of the HRD score, the proven limit value of ≥42 will be used in this study. 

In Figure 1b, we plotted the mutation frequency of all cases with *BRCA1*, *BRCA2*, or *TP53* mutations within the 30 OCs against the determined HRD score. Vertical bars connect corresponding mutation data points in a case. Figure 1b shows the regression lines of the relationship between mutation frequency and HRD score for the genes *BRCA1*, *BRCA2*, and *TP53*. All show a trend: The HRD score increases with increasing mutation frequency. A significant (*p* = 0.0156) acceptable value with R^2^ = 0.8029 is achieved exclusively for mutations in *BRCA1*. Here, it must be considered that all six *BRCA1* mutations are also associated with mutations in *TP53*, which can have an additional effect on the HRD score [42]. The two cases with the lowest mutation frequency of the *BRCA1* and *BRCA2* mutations (72-S-59 and 16-P-38) have the lowest HRD score below the cut-off value of 42. Table 2 presents the multigenic influence on the formation of the cytogenetic phenotype of HRD. In cells, gene defects can be compensated for by intact alleles in such a way that the loss of function only has a limited effect [43,44]. Thus, the frequency of gene defects in a tumor influences the gene dose effect. Figure 1a shows tumors with only one detected mutation per tumor and compares their frequency to the determined HRD score. This results in a significantly increasing linear regression (*p* = 0.0004). The higher the frequency of a pathogenic mutation, the higher the HRD score if only one mutation is present. There is a connection between the frequency of genetic changes and the influence on the HRD phenotype in genes that are involved in homologous recombination.

If there is an apparent connection between mutation frequency and the HRD phenotype, which applies particularly to *BRCA1*, the question arises whether *BRCA* mutations also repeatedly cause comparable genomic imbalances. An analysis within the 30 OCs of the retrospective study, where the most striking differences in aCGH results between mutation-carrying tumors and non-mutation-carrying tumors were analyzed, showed that in the region 10p15.3 (chr10:171262–1306517; hg19) the clearest differences can be determined (Table 3). While more than twice as many genomic gains (8/11) are found in this region in *BRCA* mutated tumors as in balanced states (3/11), in non-mutated tumors there are significantly more balanced states (15/19) than unbalanced ones (4/19). Although this difference is significant with a Chi^2^ value of 0.007, no previously known tumor-relevant genes exist in this region. The situation is similar with regard to the subordinate conspicuous loci in Table 3.

The results of aCGH do not only open the possibility of determining the phenotype of the HRD. High-resolution aCGH detects general imbalances and LOHs. Figure 2 summarizes the results of the 30 examined OC cases. Gains, losses, and LOHs of 132 tumor-relevant genes are illustrated for each tumor. In addition, gene dosage effects can influence tumor biology [44]. Just as the frequency of mutations can impact the HRD, it is also conceivable that genomic gains, losses, or LOHs of the tumor-relevant genes can impact the pathogenesis of a tumor. If diagnosing a tumor with HRD results in a therapy recommendation, it is not unreasonable to differentiate the tumors with comparable genomic scars in genes involved in the DNA repair processes. In a biaxial cluster analysis, the 30 retrospectively examined OC cases are roughly divided into 2 patient groups (clusters 1–4 and 5–6) whose genomic imbalances are reflected differently in tumor-relevant genes (Figure 2). Clusters 5–6 show uniform or hardly changed genomic doses across all gene regions. The cases in clusters 1–4 show gene groups where both genomic losses and gains predominantly occur. In the listed gene cluster block from *BRCA1* to *WRN*, with predominantly genomic losses in the OC case clusters 1–4, many genes are involved in homologous recombination with 33.9% (20/59). In this block only 6.78% of genes (4/59) are involved in NER and 5.08% of genes (3/59) are involved in MMR. The proportion of genes that are responsible for HR is significantly higher in the gene cluster (BRCA1-WRN) than in the other gene clusters. The OC cases in clusters 1–4 have another listed gene block from *PTEN* to *RECQL4*, with predominantly genomic gains, in which only 11% of genes (3/43) take on functions in homologous recombination. The proportion of genes that take on functions in NER and MMR is here at 6.98% (3/43 each), which is about the same as in the first gene block. The third listed gene block from *ATM* to *MLH3* shows a heterogeneous pattern of gains and losses in the OC case clusters 1–4. At 32.26% (10/31). This gene block again contains a comparable proportion of genes involved in homologous recombination, as in the first gene block. The collection created by biaxial clustering groups’ genomic losses has a relatively high proportion of genes involved in homologous recombination in clusters 1–4. In Figure 2 it is noticeable that in clusters 1–4 the CNV patterns of the OC cases with BRCA mutations do not differ much from those without the mutation. It is also notable that OC cases without BRCA mutations often show genomic losses in these genes. A gene dosage effect in the tumor may also have an influence on the clustering and development of the tumor. It raises the question of whether the OC case clusters 1–4 with significantly more conspicuous genomic imbalances, losses in HR genes, mutations in *TP53*, and most HRD phenotypes show a different course of the disease than the OC case in clusters 5–6. In Figure 3, the overall survival data of the retrospective OC cohort are plotted in a Kaplan–Meier curve. Accordingly, patients in OC case clusters 1–4 have a lower mean survival rate than patients in clusters 5–6. However, no statistically significant difference between the two-course curves could be calculated. No PARP inhibitors were available for any of the 30 patients in the retrospective study. For patients of the OC case clusters 1–4, with almost exclusively HRD positive phenotype, a significantly better prognosis could be indicated today with the availability of PARP inhibitors. Chromotripsis as a result of fatal DNA repair deficiencies in tumor cells opens up tremendous possibilities for treating cancer [2]. In the somatic context of tumor tissue, deficits in the HR repair mechanism of DNA cause typical genomic scars that we can identify as HRD [45]. The blockade of redundant DNA repair systems by PARPi drives the tumor cells into programmed cell death. The immense advantage of PARPi is that although they have a systemic effect on the entire organism, they do not affect healthy cells [46]. The HR system can also repair the damage DNA, despite inhibited poly(ADP-ribose) polymerase 1. Therefore, the use of PARPi in HRD-positive tumors allows a targeted, exclusive fight against the tumor cells. A reliable HRD detection is required to use this unique advantage, which we present with HRD detection using aCGH.

The application of our high-resolution aCGH to prospective OC cases to determine the HRD phenotype (with additional externally determined HRD status) shows a deviation from the externally determined HRD status in only four of 19 cases (Table 4). In two cases (Valid-07 and Valid-08), panel sequencing was inconspicuous for the *BRCA* genes, but high-resolution aCGH still revealed an HRD. With an HRD score of 43, Valid-08 is just above the limit. In two other cases (Valid-11 and Valid-14), mutations in *BRCA* genes were found, but high resolution aCGH could not confirm HRD. The Valid-11 case is also minimally below the limit with an HRD score of 41. Thus, the high-resolution aCGH test for detecting an HRD has a sensitivity of 0.77 and a specificity of 0.8. The ROC curve reflects the functionality of high-resolution aCGH for recording HRD status with an area under the curve = 0.83 (*p* = 0.003) (Figure 4). These two false-negative findings alone reduce the sensitivity to 0.77. At this point, larger numbers of cases are required to check the significance of high-resolution aCGH. In this study externally HRD assessment is used, which is partly based on detected mutations in the BRCA genes. Our data show different assessments of HRD status in a few cases, comparing the two techniques *BRCA* mutations and high-resolution aCGH. The loss of HR reflects the accumulation of genomic scars at the chromosomal level, such as LST (>10 MB), TAI (>11 MB—not crossing centromeres), and LOH (>15 MB) [20]. In our data, this phenotype is also associated in most cases with a pathogenic mutation in the *BRCA* genes. However, not always [34]. The interpolation of an HRD phenotype based on a mutation in one of the *BRCA* genes may be questioned. It is not known to what extent such a mutation can be compensated for and still allow HR. Furthermore, mutations in other HR genes can produce an HRD phenotype without having mutations in *BRCA*. Thus, the high-resolution aCGH seems to be quite suitable to determine an HRD status. A large study is needed to substantiate the effectiveness of high-resolution aCGH. 

## 4. Materials and Methods

### 4.1. Biomaterial

In this study, the HRD status of 30 ovarian tumors treated in the women’s clinic in Kiel between 1996 and 2013 was retrospectively examined using high-resolution aCGH—serving as test cohort. HRD relevant mutations from these tumors were also detected by NGS panel sequencing. To validate whether the high-resolution aCGH technique can also determine HRD phenotype prospectively, we used aCGH to further 19 ovarian carcinomas from 2016 to 2021—serving as a validation cohort. The applicability of a PARP inhibitor by HRD detection was examined externally and compared with conventional aCGH data later. The investigations are based on the vote B327/10 of the ethics committee of the medical faculty of the Christian-Albrechts-University Kiel.

### 4.2. Panel Sequencing

For panel sequencing, DNA was prepared from frozen tumor tissue using the AllPrep DNA-RNA-miRNA Universal Kit (Qiagen, Hilden, Germany, #80224). Starting from 100 ng of the tumor DNA, a library was prepared according to the DNA-Prep protocol with enrichment (Illumina, San Diego, CA, USA, #20025523 and #20025524). Hybridization detection was performed using the German HBOC TruRisk V2 panel (Illumina, San Diego, CA, USA). Sequencing was performed on Illumina NextSeq (mid-output, v2) with 2 × 150 bp paired-end reads. To calculate tumor mutation burden (TMB) as somatic mutations per 1 megabase of target region, we counted all exonic mutations in the variant allele frequency ranges 20% < VAF < 40% and 60% < VAF < 80% in order to exclude potential germline homozygous variants, potential germline heterozygous variants (Supplement Table S1). To be considered, the mutations had to be seen on forward and reverse strands. The panel size used was 144.777 kilobases.

### 4.3. aCGH

For the previous retrospective study and the subsequent validation study, according to the Agilent protocol, 1 µg of DNA from the tumor tissue and an equivalent amount of Agilent female DNA (as a reference) were processed for aCGH. In the retrospective study, 30 ovarian carcinomas were examined. For the subsequent validation study, 19 ovarian carcinomas with known status of *BRCA* mutation or known HRD status were used. DNA was digested with Alu I and RSA I restriction enzymes according to Agilent’s aCGH protocol. Then, tumor DNA was labeled with Cy5-dUTP and the reference DNA with Cy3-dUTP. The samples were purified and hybridized according to the Agilent protocol. The hybridized and washed array was recorded with a Dx microarray scanner G5761A from Agilent. The raw data were extracted using the Agilent Feature Extraction Software 3.0.5.1 (Agilent Technologies Inc., Santa Clara, CA, USA) and processed by the Agilent Cytogenomics 3.0.6.6 (Agilent Technologies Inc., Santa Clara, CA, USA) evaluation software. The chromosomal imbalances and the loss of heterozygosity (LOH) were then recorded with this software. In the retrospective study, 2 × 400 k+SNP arrays (Agilent design: 028081) were used and in the validation study a more cost-effective 4 × 180 k format (Agilent design: 086822) was selected, which maps the genes *BRCA1*, *BRCA2*, and *TP53* in high resolution and the proportion of SNP samples increased to 102907.

### 4.4. Determination of the HRD Score 

Based on the criteria of chromosomal changes published by Timms et al. in 2014 [20], losses of heterozygosity (LOH) longer than 15 MB, telomeric imbalances (TAI) longer than 11 MB, and breakpoints of large-scale transitions were evaluated (LST) of gains or losses longer than 10 MB. The LST counts the breakpoints involved, ignoring breaks up to 3 MB. The events and breakpoints that meet these criteria are summed into an HRD score for a case.

### 4.5. Evaluation of the HRD Score 

In general, a tumor with an HRD-score greater than or equal to 42 is defined as an HRD. To test whether the general limit for the HRD score can also be used with an Agilent high-resolution aCGH, a limit within our test cohort was first determined. To record the HRD score using regular Agilent aCGH, cases with mutations in *BRCA1* or *BRCA2* were defined as HRD-positive at the beginning of this study. In order to determine an HRD limit within the positive cases, the standard deviations of LOH, TAI, and LST of all positive cases were subtracted from the mean and then added up.

### 4.6. Statistics 

Kaplan–Meier survival graphs and ROC analysis were calculated with GraphPad-Prism Vers. 10.0.0 (GraphPad Softwares, Boston, MA, USA). Regression analyses were calculated with Statistica Vers. 14.0.1.25 (TIBCO Software Inc., Munich, Germany). The cluster analyses were done by Origin Ver. 9.8.0.2.00 (OriginLab Corporation, Northampton, MA, USA). Chi^2^-tests were calculated by Excel 2019 MSO (Version 1809, Microsoft, Redmond, WA, USA).

## 5. Conclusions

The determination of mutations in genes of homologous recombination alone can only show an indirect indication of the actual HRD status in patients. In principle, information about genomic scars across the entire genome is necessary to determine the HRD score [45]. As we can see, mutations in *BRCA* do not indicate HRD in all cases. Detection systems such as aCGH that can directly capture the genomic scars have advantages over systems that only interpolate the HRD through mutation detection. The high-resolution aCGH can also recognize the genomic scars directly, with a sensitivity of 0.7 and a specificity of 0.8, and is definitely suitable for recording OC tumors’ HRD status. In addition to the genomic scars, which provide such crucial therapeutic information, high-resolution aCGH can detect genomic imbalances, which may also detect cytogenetic classification. The most striking regions in the comparison between *BRCA* mutation carriers and *BRCA* wild-type tumors show no evidence of subsequent cytogenetic changes. Larger numbers of cases need to be investigated to confirm this.

## Figures and Tables

**Table 1:** The OCs that have mutations in *BRCA1* or *BRCA2* in the retrospective study.Columns with the frequency of genomic scars (LOH, TAI, and LST breakpoints) are listed next to the columns with patient code and mutations in *BRCA* genes. The HRD score for each patient is listed on the right The respective type’s average value (mean) and standard deviation (std. dev.) are shown below the genomic scars.

Patient-ID	Mutation *BRCA1/2*	LOH	TAI	LST-Break Points	HRD-Score
51-J-69	1	24	28	98	150
31-J-53	2	13	10	74	97
85-K-49	1	22	15	64	101
21-R-22	2	9	19	58	86
24-S-35	1	1	17	53	71
74-R-62	1	17	20	47	84
47-G-58	2	15	17	44	76
29-B-70	1	37	14	43	94
36-B-63	2	9	18	32	59
72-S-59	1	0	7	29	36
16-P-38	2	0	0	7	7
					
	Mean	13	15	50	
	Std. dev.	11	7	24	
	Mean–std. dev.	2	8	26	Sum 35

HRD is calculated as the sum of breakpoints in LST (>10 MB), events of TAI (>11 MB—not crossing centromeres), and events of LOH (>15 MB). At the bottom is the result of subtracting the standard deviation from the mean. The summation of these results and, thus, the determined cut-off is, on the right (sum).

**Table 2:** Results of panel sequencing and aCGH in 30 OC. Listed are OC with comparisons of the mutation descriptions in the BRCA genes and TP53. Mutations in other genes are also listed. The frequency of the mutations is shown in brackets. The HRD score is listed on the right.

Patient-Code	*BRCA1*	*BRCA2*	*TP53*	Further Genes	LOH	TAI	LST-Break Points	HRD- Score
51-J-69	c.5266_5267 insC (89%)		c.520A>T (85%)		24	28	98	150
71-T-53			c.375G>A (31%), c.764T>G (59%)		3	25	97	125
49-J-56			c.268_269delTC (68%)		24	20	66	110
85-K-49	c.1166delG (53%)		c.463A>C (36%)	*CHEK2* (42%)	22	15	64	101
31-J-53		c.5466_5467insA (64%)	c.659A>G (54%)		13	10	74	97
29-B-702	c.1687C>T (68%)		c.375+2T>A (34%)	*MLH1* (48%)	37	14	43	94
23-S-36			c.452C>A (91%)		16	20	51	87
21-R-22		c.657_658delTG (71%)	c.1013_1014insT (42%)	*MUTYH* (25%), *SLX4* (49%)	9	19	58	86
74-R-62	c.4035delA (71%)		c.659A>G (49%)		17	20	47	84
8-T-45			c.824G>T (95%)		15	21	46	82
38-B-55			c.402T>A (37%)	*RECQL* (50%), *RAD51D* (67%), *RAD51C* (31%)	20	16	43	79
42-H-37			c.524G>A (92%)		16	17	46	79
47-G-58		c.8933C>G (70%)	c.638G>T (64%)	*NF1* (30%)	15	17	44	76
24-S-35	c.2908A>T (44%)		c.224delC (44%)	*Fam175A* (62%)	1	17	53	71
25-S-37			c.814G>A (36%)	*FANCM* (43%)	21	12	9	62
36-B-63		c.4363G>T (73%)	c.949C>T (70%)		9	18	32	59
73-W-39			c.559+1G>C (59%)	*MAP3K1* (44%), *RAD50* (75%), *SMARCA4* (54%)	1	17	30	48
59-S-54			c.764_766delTCA (79%)		12	13	19	44
17-P-40			c.517G>A (84%)	*CDH1* (36%)	3	12	28	43
72-S-59	c.906delT (30%)		c.626_627delGA (28%)	*PMS2* (47%)	0	7	29	36
33-T-36			c.536A>G (66%)		10	9	6	25
45-K-67				*MSH2* (49%)	4	4	4	12
97-W-65					9	0	0	9
16-P-38		c.5164_5165delAG (56%)			0	0	7	7
99-T-47			c.626_627delGA (38%)		0	2	3	5
107-S-59				*FANCM* (49%)	1	0	2	3
92-W-69				*CDH1* (50%)	0	1	1	2
88-K-51				*MSH6* (50%)	0	0	0	0
12-L-52					0	0	0	0
54-D-39					0	0	0	0
		Mutation (Frequency)						

**Table 3:** Representation of those regions in the 30 OC cases that show the largest copy number variations (CNV) differences between *BRCA* mutation-carrying tumors and *BRCA* normal tumors.

		Cases with *BRCA 1/2* Mutation			Cases without *BRCA 1/2* Mutation			
Chr. Region (bp-Position)	Incl. Tumor Gene	No. Gains	No. Balance	No. Losses	No. Gains	No. Balance	No. Losses	Chi^2^
10p15.3 (171262–1306517)	no	8	3	0	3	15	1	0.0073
10p15.3 (1306517–3793806)	no	7	4	0	3	15	1	0.0252
11p15.4 (2976948–5785900)	no	0	5	6	1	16	2	0.0282
11p15.4 (5785900–5809417)	no	0	4	7	1	16	2	0.0086
11p15.4 (5809417–9463851)	no	0	5	6	1	16	2	0.0282
11p15.4 (9463851–9516249)	no	0	5	6	2	15	2	0.0248
11p15.4–11p15.3 (9516249–12042992)	no	0	5	6	1	16	2	0.0282

**Table 4:** HRD-score results from 19 OCs were examined prospectively to validate the high-resolution aCGH to determine the HRD-score. Comparison of the externally determined HRD status and those determined by us, using high-resolution aCGH.

Patient-Code	Extern HRD Examination	LOH	TAI	LST-Break Points	HRD- Score	HRD
Valid-01	HRD detected	18	11	63	92	yes
Valid-02	mutated *BRCA1*	5	14	61	80	yes
Valid-03	mutated *BRCA2*	15	18	36	69	yes
Valid-04	mutated *BRCA1*	18	9	32	59	yes
Valid-05	mutated *BRCA1*	9	8	38	55	yes
Valid-06	mutated *BRCA1*	0	12	43	55	yes
Valid-07	*BRCA1/2* wt	5	14	32	51	yes
Valid-08	*BRCA1/2* wt	12	9	22	43	yes
Valid-09	mutated *BRCA2*	10	17	15	42	yes
Valid-10	*BRCA1/2* wt	13	14	14	41	no
Valid-11	mutated *BRCA1*	11	16	14	41	no
Valid-12	*BRCA1/2* wt; HRD negative	0	14	26	40	no
Valid-13	*BRCA1/2* wt	0	1	23	24	no
Valid-14	mutated *BRCA2*	0	4	6	10	no
Valid-15	*BRCA1/2* wt	4	0	0	4	no
Valid-16	HRD negative	0	1	2	3	no
Valid-17	*BRCA1/2* wt	0	0	2	2	no
Valid-18	*BRCA1/2* wt	0	0	0	0	no
Valid-19	*BRCA1/2* wt	0	0	0	0	no

Positive HRD findings are shown in red.

## Data Availability

The data presented in this study are available on request from the corresponding author.

## Supplementary Materials

The following supporting information can be downloaded at: https://www.mdpi.com/article/10.3390/ijms242417467/s1.
