# Supplementary material for: Assessing the Phenotype of a Homologous Recombination Deficiency Using High Resolution Array-Based Comparative Genome Hybridization in Ovarian Cancer
Source: Int J Mol Sci. 2023 Dec 14;24(24):17467. doi: 10.3390/ijms242417467 (PMC10743768; doi:10.3390/ijms242417467)
Supplement: Supplementary file 1 [file ijms-24-17467-s001.zip › ijms-2713624-supplementary.pdf]

Supplement Table S1:

OC	TMB	LOH	TAI	LST	HRD-Score
51-J-69	27.1	24	28	98	150
71-T-53	27.1	3	25	97	125
49-J-56	13.5	24	20	66	110
85-K-49	135.3	22	15	64	101
31-J-53	67.7	13	10	74	97
29-B-70	162.4	37	14	43	94
23-S-36	67.7	16	20	51	87
21-R-22	148.9	9	19	58	86
74-R-62	175.9	17	20	47	84
8-T-45	67.7	15	21	46	82
38-B-55	203.0	20	16	43	79
42-H-37	67.7	16	17	46	79
47-G58	128.6	15	17	44	76
24-S-35	101.5	1	17	53	71
25-S-37	47.4	21	12	29	62
36-B-63	27.1	9	18	32	59
73-W-39	175.9	1	17	30	48
59-S-54	115.0	12	13	19	44
17-P-40	94.7	3	12	28	43
72-S-59	60.9	0	7	29	36
33-T-36	20.3	10	9	6	25
45-K-67	6.8	4	4	4	12
97-W-65	13.5	9	0	0	9
16-P-38	47.4	0	0	7	7
99-T-47	81.2	0	2	3	5
107-S-59	6.8	1	0	2	3
92-W-69	13.5	0	1	1	2
88-K-51	6.8	0	0	0	0
12-L-52	13.5	0	0	0	0
54-D-39	6.8	0	0	0	0

Here the calculated Tumor Mutation Burden (TMB) from panel sequencing (NGS) of all 30 retrospective analyzed OC and their HRD score determined using high-resolution aCGH are compared. Regression analysis revealed no association between TMB and HRD score ($R^2 = 0.1482$; $p = 0.0356$).
